# Research Advancements in Salt Tolerance of Cucurbitaceae: From Salt Response to Molecular Mechanisms

**DOI:** 10.3390/ijms25169051

**Published:** 2024-08-21

**Authors:** Cuiyun Chen, Wancong Yu, Xinrui Xu, Yiheng Wang, Bo Wang, Shiyong Xu, Qingkuo Lan, Yong Wang

**Affiliations:** 1 Institute of Germplasm Resources and Biotechnology, Tianjin Academy of Agricultural Sciences, Tianjin 300192, China; 2120221454@mail.nankai.edu.cn (C.C.); wancyu@163.com (W.Y.); 2120221518@mail.nankai.edu.cn (X.X.); sywyhyx@126.com (Y.W.); wangbo0426mm@163.com (B.W.); whui175@163.com (S.X.); 2 College of Life Sciences, Nankai University, Tianjin 300071, China; 3 State Key Laboratory of Vegetable Biobreeding, Tianjin Academy of Agricultural Sciences, Tianjin 300192, China

**Keywords:** salt stress, SOS, transcription factors, Cucurbitaceae, molecular mechanisms

## Abstract

Soil salinization severely limits the quality and productivity of economic crops, threatening global food security. Recent advancements have improved our understanding of how plants perceive, signal, and respond to salt stress. The discovery of the Salt Overly Sensitive (SOS) pathway has been crucial in revealing the molecular mechanisms behind plant salinity tolerance. Additionally, extensive research into various plant hormones, transcription factors, and signaling molecules has greatly enhanced our knowledge of plants’ salinity tolerance mechanisms. Cucurbitaceae plants, cherished for their economic value as fruits and vegetables, display sensitivity to salt stress. Despite garnering some attention, research on the salinity tolerance of these plants remains somewhat scattered and disorganized. Consequently, this article offers a review centered on three aspects: the salt response of Cucurbitaceae under stress; physiological and biochemical responses to salt stress; and the current research status of their molecular mechanisms in economically significant crops, like cucumbers, watermelons, melon, and loofahs. Additionally, some measures to improve the salt tolerance of Cucurbitaceae crops are summarized. It aims to provide insights for the in-depth exploration of Cucurbitaceae’s salt response mechanisms, uncovering the roles of salt-resistant genes and fostering the cultivation of novel varieties through molecular biology in the future.

## 1. Introduction

**Figure 1:**
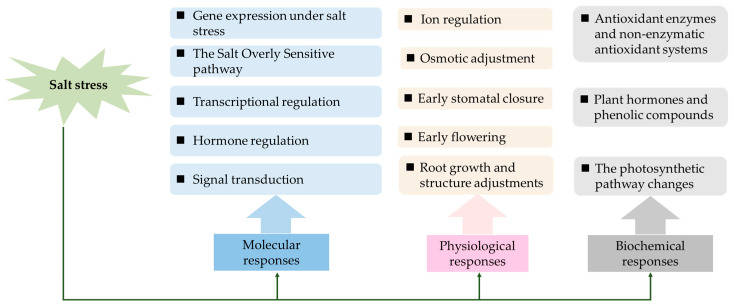
A model summarizing the regulation mechanism of salt stress in plants. The different colors in the diagram represent distinct types of responses to salt stress: Blue shapes correspond to Molecular responses. Pink shapes correspond to Physiological responses. Gray shapes correspond to Biochemical responses.

Soil salinization has become a global obstacle that impedes plant growth and endangers food security [1]. UNESCO reports indicate that approximately one billion hectares globally are affected by salinization, and it is projected that, by 2050, salinization will impact 50% of the arable land, thereby posing substantial risks to the future food supply [2]. The exacerbation of saline soils results from both global climate change and human activities, such as excessive chemical use and poor irrigation practices. These factors contribute to the steady expansion of affected areas [3]. In response to deteriorating soil conditions, plants have developed complex physiological, biochemical, and molecular mechanisms to cope with different levels of salt stress (Figure 1) [4,5]. Physiologically, these adaptations include (1) ion regulation and compartmentalization [6]; (2) the synthesis of compatible solutes or osmotic adjustment substances, like proline, sugar alcohols, and anthocyanins; (3) early flowering or early stomatal closure [7]; and (4) alterations in root development [8]. Biochemically, plants respond through (1) the activation of antioxidant enzyme and non-enzymatic antioxidant systems, such as glutathione and carotenoids, which help protect against oxidative stress [9]; (2) the production of plant hormones and phenolic compounds; and (3) adjustments in the photosynthetic pathway [5]. Molecular mechanisms under salt stress include: (1) gene expression, involving genes related to ion transporters, compatible solute synthesis, hormone synthesis, and free radical scavenging enzymes [4,10]; (2) the SOS pathway [11]; (3) transcriptional regulation, such as the WRKYs and NAC TF family [12]; (4) hormone regulation [13]; and (5) signal transduction, such as the Ca^2+^ signaling pathway [14] and the MAPK cascade signaling pathway [15]. As shown, there are two or more levels of adaptation to salt stress. Salt reaction causes physiological, biochemical, and molecular changes, which can be used to maintain plant homeostasis and alleviate salt stress.

Cucurbitaceae, encompassing crops like melon (*Cucumis melo*), watermelon (*Citrullus lanatus*), cucumber (*Cucumis sativus*), pumpkin (*Cucurbita moschata*), loofah (*Luffa cylindrica*), and zucchini (*Cucurbita pepo*), are not only staples in our diet with significant economic value [16] but are also notably sensitive to a series of biotic and abiotic stresses, particularly salt stress [11,17]. Presently, soil salt stress represents a critical abiotic factor curtailing the yield of economically important crops such as Cucurbitaceae. Concentrating research on plants’ response pathways to salt stress and external improvement techniques holds remarkable potential for enhancing plant salt tolerance, boosting crop yield, and improving crop quality [18]. Accordingly, this focus has become a primary research interest for many plant physiologists, biochemists, molecular biologists, geneticists, and breeders. Moreover, given the escalating severity of soil salinization, enhancing the use of saline-alkali land and investigating molecular regulatory mechanisms and metabolic pathways for plant salt tolerance carry significant theoretical and practical implications for the cultivation of saline-alkali land and the breeding of salt-resilient crops [19].

The plant response to salt stress encompasses nearly every facet of plant physiology and metabolism, thereby rendering the salt response signaling network exceptionally intricate [20]. This article seeks to deliver an exhaustive overview of the detrimental results of salt stress on Cucurbitaceae plants, their responses, the SOS pathway, and the current research status of salt tolerance mechanisms in economically important crops, like cucumber, watermelon, and loofah. Delving into the similarities between Cucurbitaceae plants’ salt response mechanisms and the salt tolerance traits of other plants is paramount. Understanding the Cucurbitaceae salt response network, uncovering salt-resistant genes, and fostering the development of new varieties through molecular biology in the future hold significant importance.

## 2. Salt Stress Detriment and Response

### 2.1. Salt Tolerance and Hazards of Different Cucurbitaceae

Research indicates that salt stress profoundly impacts the growth and development of Cucurbitaceae [21]. Salt stress affects Cucurbitaceae plants’ water absorption, cell elongation, lateral branch development, seed germination, photosynthesis rate and nutrient absorption and adversely impacts overall plant growth [22]. Since, the high concentration of sodium and chloride ions in saline soils creates an environment of high osmotic pressure, limiting the uptake of water and nutrients by Cucurbitaceae crops cells and contributing to the accumulation of excessive salt ions intracellularly [23]. This results in stunted seedlings, chlorosis, underdeveloped root systems, and even yellowing, wilting, and, in extreme cases, plant death [11].

**Figure 2:**
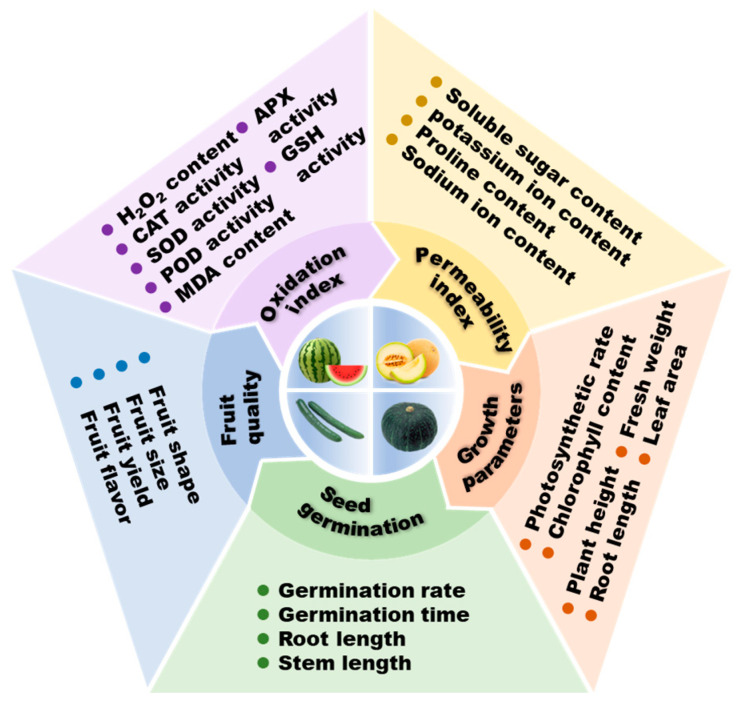
Common salt tolerance evaluation indexes of Cucurbitaceae.

Cucurbitaceae contains a variety of species, and the sensitivity to salt varies significantly among different species. The salt tolerance of different germplasm materials also showed some differences among the same species [24]. Numerous investigations conducted have shown that the stress effect is closely related to factors such as the intensity and length of stress, as well as the characteristics of the plant itself [25]. The variation in salt tolerance among different species of Cucurbitaceae can be attributed to several factors, including specific physiological mechanisms such as ion homeostasis and osmotic adjustment, unique gene expression and regulatory patterns, distinct morphological structures, and adaptations to different ecological niches such as coastal areas, saline soils, and diverse geographic distributions. These factors collectively contribute to the diverse and complex nature of salt tolerance within Cucurbitaceae [6,26,27]. Together, these differences lead to the diversity and complexity of salt tolerance among different genetic resources of Cucurbitaceae [25,28,29,30]. At present, it is believed that, among the Cucurbitaceae plants, pumpkin and melon have a better salt tolerance; loofah, watermelon, and bitter gourd have a relatively high sensitivity to salt; and cucumber has the highest sensitivity to salt stress [31]. Next, we will illustrate salt sensitivity between different species based on early salt tolerance assessments for several species such as cucumber, pumpkin, watermelon, and melon. Important evaluation indexes of salt tolerance are shown in Figure 2.

Cucumber ranks among the most widely cultivated vegetables worldwide. However, cucumbers are sensitive to salt stress, with young seedlings in saline soils exhibiting significant and reduced growth indicators [21]. In investigating the salt tolerance of seed germination, salt stress not only delayed the germination rate of cucumber seeds but also greatly diminished the germination rate and stem length of cucumber seeds at salt concentration (200 mM) [32]. In addition, root length was significantly reduced for all treatments. However, the fresh weight of cucumber increased at lower levels of salt stress (25 and 50 mM NaCl), but decreased significantly at higher levels, and the dry weight decreased significantly at all levels of salt stress [25]. In the evaluation of salt tolerance in different cucumber genetic germplasms, 100 mM NaCl and 150 mM NaCl were usually used as suitable concentrations for assessing salt tolerance in seedlings [26]. At 100 mM treatment, most of the germplasm showed salt damage traits, and under 150 mM NaCl treatment, all cucumber seedlings showed salt damage traits, such as leaf wilting, yellowing, and death [24].

Watermelon also has a relative tolerance to salt stress; so, it serves as an excellent model crop for studying salt stress-induced responses [33]. Levels of 120 mM NaCl can reflect the degree of salt damage among watermelon germplasm resources with different salt tolerance abilities, indicating their salt tolerance at the germination stage [34]. Additionally, 150 mM NaCl is often used in salt stress experimental treatments for watermelon seedlings [6]. Under these conditions, the biomass and growth potential of watermelon seedlings decrease significantly [34]. Early studies reported that, at salt concentrations greater than 75 mM NaCl, the growth of watermelon seedlings will be inhibited. Moreover, the stress levels in watermelon seedlings intensified with the rise in salt concentrations, and when the salt concentration was greater than 150 mM NaCl, seedling expansion and maturation were significantly impeded, which drastically diminished the production and quality of watermelon. A study on watermelon seedlings treated with 150 mM NaCl revealed that salt stress markedly decreased biomass and K^+^ levels in roots and leaves, while significantly elevating Na^+^, Cl^−^, and MDA contents. Notably, salt-tolerant varieties exhibited higher physiological indicators, such as K^+^ accumulation, Cl^−^ levels, and MDA content, compared to salt-sensitive varieties [6].

Although melon can be cultivated in saltpans due to its moderate salt tolerance, there is a risk of soil salinization during production [35]. Prior research has assessed the ability of various melon cultivars to withstand salt throughout the seed and seedling phases. Curiously, melons with thicker skins can withstand more salt than those with thinner skins [30]. The concentration of melon salt treatment is usually about 200 mM NaCl. Low concentrations of NaCl stress have been shown to enhance the osmotic regulation of cells and facilitate melon seed germination; however, as NaCl concentration rises to 100 mM, melon seed germination is severely inhibited, leading to a reduction in seed germination potential, germination index, and vigor index, as well as a longer germination period [25]. According to [36,37,38,39], and other studies, melon seeds under salt stress showed a reduction in coaxial length, radicle length, and fresh weight as the concentration increased. Additionally, the inhibitory effect gradually increased as the stress time extended. Interestingly, not all outcomes of salt stress on melon are negative. In field salinity experiments, the thickness of the fruit may not be affected by salt concentration, while the sweetness of the fruit can increase due to the reduction in fruit size and the increase in sugar transport by the phloem caused by moderate salinity [25,38]. On the other hand, melon’s physiological indices and growth parameters declined as the salt concentration increased. According to [30], there was a considerable decrease in the leaf relative water content, pigment content, stomatal density, leaf area, biomass, and K^+^ concentration in leaves and stems, and leaf and stem K^+^/Na^+^ ratio. This finding aligns with previous research on cucumbers, rice, and sorghum. Furthermore, salt stress affected not only fruit output, but also some fruit physical characteristics, such as size and pulp percentage [36,37,38,39].

Another prominent member of the Cucurbitaceae family of vegetables is the pumpkin, which is more resistant to salt than other Cucurbitaceae species. If NaCl was used to stress pumpkin at a concentration of 120~240 mM, the changes in growth indexes or physiological and biochemical indexes were significantly different among different pumpkin germplasm materials [40]. Due to its greater salt tolerance, it is often used as a grafted rootstock for other family members, including cucumbers, watermelons, and melons [41,42]. Tarchoun assessed the salt tolerance of 15 native varieties of Tunisian pumpkin by utilizing 12 agronomic characteristics, including germination rate, shoot length, root length, and fresh weight, as well as 3 biochemical traits, MDA, proline, and chlorophyll [31]. The feasibility of selecting salt-tolerant pumpkin germplasm early on based on germination and seedling growth potential under salinity stress was presented in this work. Huang et al. [43] provided a solid scientific foundation for the breeding of Cucurbitaceae. They also contributed significantly to understanding the molecular mechanisms underlying the variation in salt tolerance between cucumber and pumpkin, suggesting that the latter’s high accumulation of H_2_O_2_ at the root tip and the former’s strong K^+^ absorption capacity play key roles. In addition to these cucurbit crops, people also frequently eat loofah [3], bitter gourd [44], and zucchini [45], yet there is little research on the salt tolerance of these crops.

### 2.2. Salt Response of Cucurbitaceae from a Physiological Point of View

The excessive accumulation of Na^+^ and Cl^−^ can interfere with the absorption of K^+^, Ca^2+^, and other vital nutrients, contributing to salt toxicity in Cucurbitaceae and many other plants [6]. High concentrations of Na^+^ can also disrupt cellular osmotic balance, compromise membrane functions, and increase reactive oxygen species (ROS), thereby adversely affecting normal plant growth [11,17,46,47,48]. Additionally, Cl^−^ can disrupt cell membrane systems and organelle structures, leading to a decline in chlorophyll content and hindering the photosynthesis of Cucurbitaceae [31]. Thus, preserving the intracellular Na^+^/K^+^ balance is the main embodiment of the salt tolerance mechanism of Cucurbitaceae. As the research deepened, scientists found that, under salt stress, Cucurbitaceae can expel sodium from the roots, or absorb sodium, transport it to the aerial parts and sequester in vacuoles [37,49]. The capability to sequester sodium and sustain a high Na^+^/K^+^ ratio has been shown to be a significant feature of tolerant melon cultivars in both seedling studies [25] and field trials [38,50].

To address osmotic imbalance, Cucurbitaceae can synthesize some macromolecular organic compounds, such as sugars, alcohols, polyamines, etc., to improve the osmotic potential of cells and improve the water absorption capacity under high salt conditions [51]. In addition to synthesizing organic matter, ion compartmentalization can also alter the osmotic potential of cells and improve salt tolerance in Cucurbitaceae [52]. Under salt stress, cucumber mainly absorbs K^+^ from the environment into cells through the potassium ion transporter CsAKT1, while pumpkin absorbs K^+^ from the environment into cells through the potassium ion transporter HAK5, thereby increasing intracellular osmotic pressure [43,53]. For Na^+^, on the one hand, Cucurbitaceae reduces the absorption of Na^+^ and increases the efflux of Na^+^ through the high-affinity sodium ion /potassium ion transporter HKT, and on the other hand, the Na^+^ that enters the cytoplasm is transported into the vacuole by the Na^+^/H^+^ antiporter (NHX) located on the vacuolar membrane. This transport process helps to create a high osmotic potential within the vacuole compared to the cytoplasm and apoplast, thereby maintaining a normal metabolic activity in the cell [54,55]. Among them, the regulation of pumpkin CmoHKT1;1 [56] and melon CmHKT1;1 [55] activity in response to salt stress has been well studied. Additionally, the plasma membrane Na^+^/H^+^ antiporter SOS1 can promote Na^+^ efflux, helping to maintain Na^+^/K^+^ balance and osmotic balance. Different from the above studies, Zhang et al. found an inward Shaker K^+^ channel in salt-tolerant melon varieties, MIRK (melon inward rectification K^+^ channel) [57], and also demonstrated that external Na^+^ can inhibit the activity of MIRK channels.

Previous studies have also demonstrated that halophytes regulate stomatal openings to mitigate moisture loss and wilting. A comparable mechanism has been observed within the Cucurbitaceae family. In grafting experiments, it was observed that cucumbers grafted onto pumpkins exhibited rapid stomatal closure, facilitating adaptation to osmotic stress caused by salinity [7]. Salt stress can also impact root development in plants, prompting the formation of adventitious roots that enhance nutrient and water absorption capacity. Research indicates that conventional plant hormones like ethylene [58] and gaseous molecules such as hydrogen sulfide [59] serve as signaling agents involved in adventitious root development under salt stress in cucumbers.

### 2.3. Salt Response of Cucurbitaceae from a Biochemical Point of View

Similar to heat and drought stresses, salt stress frequently induces an excessive accumulation of ROS in cucumbers [52], watermelon [60], melons, pumpkins [30], and loofahs [61]. ROS such as O_2_^−^, H_2_O_2_, and OH^−^ are known to damage cell membranes via lipid peroxidation, disrupt DNA strands, deactivate essential enzymes, and disturb redox homeostasis, severely impairing plant growth [62]. To mitigate ROS accumulation, plants have developed antioxidant defense systems that include both enzymatic and non-enzymatic components.

Numerous experiments have demonstrated a significant increase in the activity of antioxidant enzymes such as SOD, CAT, and POD in Cucurbitaceae seedlings under salt stress, contributing to the maintenance of redox homeostasis and reduction in salt toxicity [60]. Non-enzymatic antioxidants like carotenoids, glutathione, and ascorbic acid not only directly scavenge ROS to mitigate oxidative stress but also serve as substrates within ROS scavenging systems [1]. Consequently, these substances often serve as indicators of salt tolerance.

Salt stress exerts significant effects on plant photosynthesis [62]. Stomatal closure induced by salt stress reduces water loss but also diminishes the intercellular CO_2_ concentration within leaves. Consequently, this diminishes NADPH availability to the Calvin cycle, constrains chlorophyll synthesis and Rubisco activity, and impairs the photosynthetic electron transport chain. Importantly, the suppression of the photosynthetic electron transport chain by salinity also fosters the excessive accumulation of ROS. This ROS buildup further accelerates chlorophyll degradation, decreases the photochemical efficiency of photosystem II (PSII), creating a detrimental feedback loop [63].

Furthermore, plant hormones and phenolic compounds have demonstrated protective benefits for biological systems under salt stress conditions [64]. The levels of polyphenols and phenols in cucumbers significantly increase under salt stress [32]. Additionally, salt stress promotes the accumulation of osmotic regulators such as soluble sugars and proline, which significantly reduces MDA content and relative leaf conductivity in Cucurbitaceae crops [65].

## 3. Research on Salt Stress at the Molecular Level

### 3.1. Gene Expression in Response to Stress

**Figure 3:**
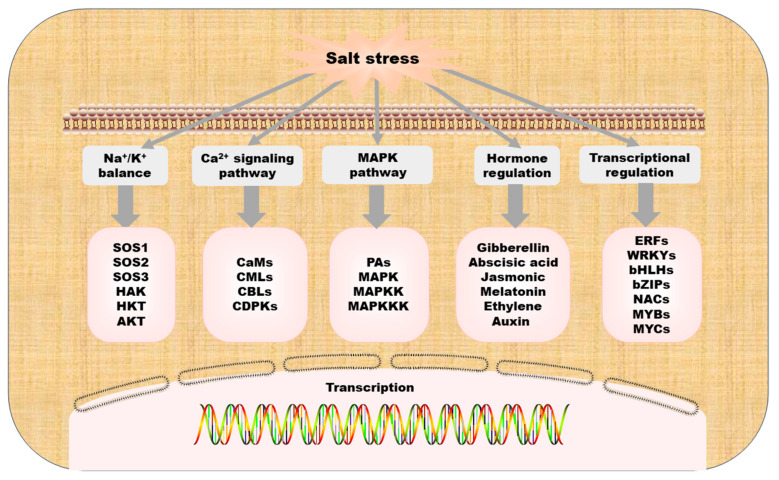
A model summarizing the molecular regulation mechanism in Cucurbitaceae.

Cucurbitaceae is a vital cash crop, making it essential to understand the regulatory mechanisms of its salt tolerance. The main molecular regulation mechanism of Cucurbitaceae is shown in Figure 3. Through the cloning and analysis of candidate genes in the recombinant inbred line population produced by crossing salt-tolerant and salt-sensitive line, it was found that salt tolerance in cucumber seedlings is a quantitative trait governed by multiple genes, aligning with findings in watermelon studies [6], melon [39], and other species. In addition, qST6.2, an important locus regulating salt tolerance in cucumber seedlings, was identified, which paved the groundwork for the precise mapping of cucumber salt tolerance genes [29]. Subsequently, 220 cucumber materials enabled genome-wide association study (GWAS) and salt tolerance gene identification, which further verified the complexity of cucumber salt tolerance genes, and the expression of salt tolerance genes was variable among different germplasms and species, which provided an effective basis for subsequent research on cucumber seedling salt tolerance genes and molecular mechanisms [28].

The expression of various related genes in Cucurbitaceae under salt stress enhanced the salt tolerance of Cucurbitaceae plants through different response modes [30]. First, under salt stress, specific transcription factors, such as DREB, bZIP, and NAC, bind to cis-regulatory elements like DRE, ABRE, and NACR, respectively, activating the expression of downstream target genes involved in osmotic adjustment, ion homeostasis, and antioxidative defense, thus enhancing salt tolerance [66]. Second, salt tolerance genes such as *HKT1;1*, *SOS1*, and *NHX1* are transcribed and translated into proteins that function in sodium exclusion, sequestration, and compartmentalization, directly contributing to the improvement in plant salt tolerance [55]. Third, the expression of salt-responsive genes is modulated by complex gene networks and signaling pathways, where key regulatory genes interact with each other through feedback and cross-talk mechanisms, fine-tuning the overall salt stress response and adaptation in plants [67]. These salt-tolerant genes can be broadly divided into five categories: those involved in ion balance, those involved in the regulation of reactive oxygen species, those involved in transcriptional regulation, those involved in hormone regulation, and those involved in signal transduction [68]. However, some genes may respond to salt stress by mediating multiple regulatory types, such as circular RNAs (circRNAs) [69]. In recent years, some reports have indicated circRNAs are also vital in salt stress responses in plants, such as tomatoes [70] and maize [71]. However, at present, only circRNAs in cucumber have been identified and characterized in Cucurbitaceae, which may be involved in the corresponding pathways of transcription, signal transcription, cell cycle, metabolic adaptation, and ion homeostasis in response to salt stress [72]. Over the years, significant progress has been achieved in understanding salt tolerance genes for Cucurbitaceae salt stress, and more and more, salt tolerance genes have been identified and verified, especially for cucumber, as shown in Table 1.

### 3.2. The Salt Overly Sensitive Pathway

The toxicity of saline-alkali soil to plants primarily stems from sodium ions. An imbalance in sodium ions directly affects the normal growth of plant cells, making the maintenance of sodium ion homeostasis crucial for enhancing plant salt tolerance [95,96]. Research has demonstrated that the SOS pathway is a unique route by which plants extrude sodium ions from cells under salt stress [11,17,46,47,48]. The discovery of the SOS signaling pathway has been particularly pivotal for researching salinity tolerance mechanisms [97]. In this pathway, the calcium-binding protein SOS3 detects cytoplasmic calcium signals brought by salt stress and cooperates with the Ser/Thr protein kinase SOS2, leading to its activation [47,98]. The activated SOS2 enhances the plant’s salt resistance by phosphorylating and activating the SOS1 protein, which facilitates the extrusion of Na^+^ [99,100]. Beyond these core proteins, molecules such as phosphatidic acid and polyamine [10] are also intimately connected to the SOS pathway’s role in enhancing plant salt tolerance. However, the mechanism of SOS regulation in Cucurbitaceae is rarely reported. Importantly, the SOS2 protein is located mainly in the plasma membrane of cucumber, and polyamines can induce the expression of the SOS2 gene family in cucumber under salt stress [101].

### 3.3. Transcriptional Regulation

The functional analysis and validation of salt-related transcription factors using comprehensive approaches like whole-genome identification and transcriptome sequencing have become more accessible. In recent studies, multiple TFs have been identified as playing a crucial role in the salt stress responses of Cucurbitaceae by regulating the transcription of several genes. In general, they act as crucial positive regulatory signaling molecules in the salt stress response [102]. The genetic identification and analysis of the transcription factors of the watermelon homology domain leucine zipper (HD-ZIP) under abiotic stress found that the expression of most of these ClHDZs was induced by salt stress, especially *ClHDZ20* and *ClHDZ36* in HD-ZIP I and *ClHDZ1* and *ClHDZ18* in HD-ZIP II [103]. Thirty-six *ClDof* genes were identified in the single-finger DNA binding (Dof) family of watermelon plants, which may play a regulatory role under salt stress [104]. Additionally, RNA sequencing (RNA-seq) was used to detect changes in gene expression in watermelon seedlings under short-term salt stress, discovering a variety of transcription factors, including members from the ERF, WRKY, NAC, bHLH, and MYB families being overexpressed, providing new perspectives for the mechanisms of watermelon salt tolerance [105]. Furthermore, RNA-seq analysis was conducted on the roots and leaves of two watermelon varieties, one salt-sensitive and the other salt-tolerant, and revealed many transcription factors, including AP2-EREBP, bZIP, bHLH, MYB, NAC, OFP, and TCP, and WRKY, that are closely connected with salt tolerance-related genes, suggesting their likely involvement in the salt stress regulatory network [6].

Considerable genetic evidence has underscored the indispensable role of ABFs, WRKYs [83], MYBs [106], NACs [107], the basic region/leucine zipper motif (bZIP) [108], and bHLHs [106] in regulating the salt stress tolerance of Cucurbitaceae plants. The WRKY transcription factor family, one of the largest in vascular plants, plays a critical role in plant development and stress responses, including salt stress [66]. Multiple studies have revealed that NaCl treatment in cucumber [83], watermelon [6], and zucchini upregulates most of the WRKY gene. In particular, transcriptome analysis showed that a variety of WRKY transcription factors, such as *CsWRKY27*, *CsWRKY41*, and *CsWRKY50*, responded to cucumber salt stress [83]. Furthermore, the overexpression of the *WRKY* gene from cucumber in Arabidopsis using a transgenic approach has validated the functional role of WRKY TFs in enhancing salt tolerance.

Similarly, ERFs are involved in a wide range of stress responses, including salinity. Salinity stress enhances ethylene biosynthesis and activates downstream networks and the expression of *ERFs*. It is known that the response of ethylene in plant salt stress remains a topic of debate, but ERFs generally play a positive role in plant salt stress. For example, the five ethylene receptors ETR1, ERS1, ETR2, ERS2, and EIN4 of *Arabidopsis* are negative regulators of ethylene signaling pathways, but play a positive regulatory role in salt tolerance [45]. Consistent with *Arabidopsis thaliana* results, the Cucurbita pepo ethylene receptors *CpETR1B*, *CpETR1A*, and *CpETR2B* play an active role in salt tolerance during both the germination and vegetative growth stages [45]. In related studies of cucumber, ethylene may also be used as a subsequent signaling molecule of Ca^2+^, maintaining Na^+^/K^+^ homeostasis by enhancing the transcription and activity of Na^+^/H^+^ antiporters and H^+^-ATPases, and maintaining the integrity of the cucumber explant cell ultrastructure under salt stress [58]. In addition, transcriptome studies of cucumbers, melons, etc. also demonstrated a substantial increase in *ERF* expression levels under salt stress conditions, indicating the important role of ERF in the mechanism of plant salt response. Despite their importance, there is still very little information about the role of ERF transcription factors in the plant, with a few exceptions.

The response of NAC transcription factors in Cucurbitaceae salts has also attracted attention [109]. In pumpkin, it was suggested that *CmoNAC1* regulated root ABA and H_2_O_2_ signaling under salt stress. Subsequent root transformation experiments and RNA-seq analysis verified that *CmoNAC1* enhances squash salt tolerance by regulating *CmoRBOHD1* and *CmoNCED6* to promote H_2_O_2_ and ABA production and interacting with the promoters of *CmoAKT1* and *CmoHKT1* to modulate K⁺/Na⁺ homeostasis [107]. A genome-wide analysis was conducted to identify and characterize the response of the cucumber alkaline helix–ring–helix family under salt stress, and the study identified the *CsbHLH041* gene as a key regulator in enhancing the salt tolerance of cucumber seedlings [81].

### 3.4. Hormone Regulation

Auxins (IAA), gibberellins (GA), jasmonic acid (JA), abscisic acid (ABA), ethylene (ETH), brassinosteroids (BRs), and melatonin have all been demonstrated to play crucial roles in the response of Cucurbitaceae plants to salt stress [10,13,110]. Transcriptome analysis has identified a substantial number of genes involved in cellular metabolism and redox processes during melon salt stress. These genes are notably enriched in IAA, cytokinin, ABA, and BRs hormone signal transduction pathways [66]. Research indicates that plant auxin levels decrease under salt stress, accompanied by a reduction in the expression of auxin transport proteins [111]. Additionally, ABA has been increasingly recognized for its critical role in regulating seed germination, seedling growth, and responses to a variety of abiotic stresses [112,113].

Ethylene functions as a crucial phytohormone involved in regulatory mechanisms under abiotic stress [8]. In cucumbers, Ca^2+^ plays a pivotal role in facilitating adventitious root development under salt stress by modulating endogenous ethylene synthesis and activating ethylene signal transduction pathways [58]. Some researchers argue that ethylene acts as an adverse regulator of salt tolerance during both germination and vegetative growth stages. However, Reda et al. demonstrated that salt stress induces the upregulation of genes linked to Ca^2^⁺ signaling (*CPCRCK2A* and *CPCRCK2B*) and ABA biosynthesis (*CPNCED3A* and *CPNCED3B*), suggesting that the ethylene receptor function in the response of zucchini to salt stress may be mediated through the Ca^2^⁺ and ABA signaling pathways [74].

Melatonin acts as an essential free radical neutralizer and antioxidant in plants, promoting the enhancement in the antioxidant system under salt stress conditions. In cucumbers, melatonin regulates root development via a reactive oxygen species system, oxidases, and specific transcription factors during salt stress [114]. Notably, research on watermelon indicated that ClCOMT1, a crucial enzyme in melatonin biosynthesis, is upregulated during salt stress, leading to increased melatonin levels that enhance salt tolerance in watermelon [115]. Furthermore, melatonin might also regulate the expression of genes participating in the salt stress response [116].

### 3.5. Signal Transduction

Calcium ions are at the heart of the signaling pathway [14]. Under salt stress, Ca^2+^ acts as a secondary messenger, transmitting extracellular signals to the intracellular environment and participating in various pathways such as ROS and hormone signaling pathways. Salt stress activates cytosolic calcium, triggering phosphorylation cascades of calcium-dependent proteins or calcium sensors, including calmodulins (CaMs), calmodulin-like proteins (CMLs), calcineurin B-like proteins (CBLs), and Ca^2+^-dependent protein kinases (CDPKs), thereby activating physiological reactions to salt stress in plants [117,118,119]. It was reported that salt stress treatment could upregulate the expression of *CmCBL1* and *CmCBL3* in the melon CBL family, and the overexpression of *CmCBL1* in wild-type *Arabidopsis* could enhance plant salt tolerance to a certain extent, but the overexpression of *CmCBL3* would reduce the seed germination rate [120]. In addition, the ectopic expression of *CmCML13* in *Arabidopsis thaliana* not only improved salt tolerance during seed germination, but also enhanced the salt tolerance of transgenic *Arabidopsis* plants by significantly reducing the aboveground Na^+^ content, which operated independently of the HKT1-related pathway. The calcium signaling protein CsCDPK6 in cucumber has also been functionally characterized [84]. It was found that the ectopic overexpression of *CsCDPK6* in tobacco enhanced salt tolerance. In addition, the expression of *CsCDPK6* in the cotyledons of cucumber seedlings was inhibited by virus-induced gene silencing (VIGS) under salt stress, which also enhanced salt stress [84]. At present, it has been found that *CsCDPK6* and *CsSAMS1* interact to improve salt tolerance, but the downstream molecular mechanism needs to be further studied. Like Ca^2+^, gas signaling molecules such as nitric oxide (NO) and hydrogen sulfide (H_2_S) are also considered to be important second messengers in plants, actively participating in the signal transduction of salt tolerance in Cucurbitaceae, and their possible roles will be mentioned in subsequent chapters [59,121].

The mitogen-activated protein kinase (MAPK) cascade is another signal transduction module present in plants that also responds positively to salt stress, e.g., MKK7, *MKK9, MPK6* [122], and *OsMKK1* in rice [123]. The MAPK cascade includes three protein kinases, MAPK kinase kinase (MKKK or MAPKKK), MAPK kinase (MKK or MAPKK), and mitogen-activated protein kinase (MPK or MAPK), which are activated by extracellular stimuli and mediate signal transmission into the cell [124,125]. At present, the genes of the MAPK cascade family in the Cucurbitaceae family have been partially characterized. Through the genome-wide identification of the mitogen-activated protein kinase (MAPK) cascade and the analysis of the expression profile of *CmMAPKs*, the evolutionary homology between melon and *Arabidopsis thaliana* was revealed, and its chromosomal localization characteristics were similar to those of watermelon and cucumber, and its homologous *CmMAPK3* and *CmMAPK7* could be used as the focus of future research on salt tolerance genes [126]. The expression of 10 *CLMPKs*, such as *ClMPK1* and *ClMPK3*, and *3 CLMKKs*, such as *ClMKK2-2* and *ClMKK3*, in watermelon has also been verified in response to salt stress [125]. Moreover, exposure to abscisic acid and jasmonate significantly influenced the expression levels of certain *CsMAPK*, *CsMAPKK*, and *CsMAPK* in cucumber, suggesting that the MAPK cascade may be involved in the regulation of plant hormone networks [15]. Unfortunately, the regulatory mechanism of MAPK cascade in the downstream pathways of Cucurbitaceae is still poorly understood.

## 4. Measures to Improve Salt Tolerance of Cucurbitaceae Plants

Numerous studies report that the application of grafting [127] and exogenous substances, such as silicon [128], hormones (melatonin), nanoparticles (Se) [129], polyamines [129], and gaseous signaling molecules (NO, H_2_S) [52,130], can alleviate the effects of salt stress to a certain extent, as seen in Table 2.

### 4.1. Nanomaterials

Under salt stress, some nanomaterials, such as cerium oxide nanoparticles (CeO_2_), have been found to elevate cucumber seedling salt tolerance by modulating the antioxidant system [131]. Regarding the reasons why silicon improves cucumber salt tolerance, on the one hand, silicon elevates the hydraulic conductivity of the root system, improving the seedling moisture content, and on the other hand, silicon stimulates the accumulation of polyamines to reduce the Na^+^ content and alleviate ion toxicity [128,132]. It has been reported that the cucumber potassium ion transporter (*CsAKT1*) is vital for improving salt tolerance in cucumber using the CRISPR-Cas9 line through polyacrylic acid-coated nanoceramics (PNCs) [53]. A report indicated that researchers successfully induced multiple defenses and secondary metabolism-related transcripts of bitter gourd seedlings under salt stress by administering synthetic biodegradable selenium–chitosan nanoparticles (Se-CS NPs) [133]. Se-CS NPs not only significantly improved the physiological and biochemical parameters of bitter gourd seedlings under salt stress, but also were closely linked with the expression of salt stress defense genes [133].

### 4.2. Graft

Grafting, as a means to enhance cucumber and melon [134] salt tolerance, has also received extensive attention and has been successfully used in vegetable production. At present, the mechanism of grafting to enhance the salt tolerance of Cucurbitaceae has also been analyzed [135,136,137]. In 2018, it was proposed that pumpkin-grafted cucumber plants relied on the mechanism of increased salt tolerance produced by the outbreak of oxidase homology-dependent H_2_O_2_ through root respiration and also enhanced the elimination of Na^+^ from roots and promoted the early closure of stomata [41]. The following year, Zhang et al. again demonstrated that cucumbers grafted with pumpkin increased cucumber sensitivity to ABA and stimulated early stomatal closure, thereby improving osmotic tolerance under NaCl stress [7]. Meanwhile, the latest studies have shown that using loofah as rootstock can reduce sodium transport to the aerial parts, thereby enhancing the salt tolerance of grafted cucumber plants and improving the yield and quality [3]. This is similar to the studies in cucumber grafting with pumpkin [135,136,137] and watermelon grafting with gourd [135,136,137]. In grafting experiments, the biomass yield of melon was diminished by a salt concentration of 80 mM NaCl, but the corresponding diminution was greater in non-grafted plants with 80 mM NaCl [49]. However, the melon scion genotype did not significantly influence the plant’s response to salinity, but was between ungrafted, self-grafted, and interspecific grafted plants.

### 4.3. Polyamines

Polyamine, particularly spermidine (Spd), has emerged as a pivotal enhancer of salt tolerance in Cucurbitaceae plants [129,138]. Widespread research has underscored its significance in regulating plant physiological and biochemical pathways under saline–alkali stress [139,140]. A recent investigation has demonstrated that the application of exogenous Spd triggers the expression of Rboh genes, leading to the generation of H_2_O_2_. This, in effect, serves as a signaling molecule for the subsequent expression and activation of autophagy-related ATG genes. Subsequently, the enhanced autophagosome activity facilitates the breakdown of insoluble ubiquitinated protein aggregates, which accumulate due to salt stress damage, thereby bolstering the salt tolerance of cucumber seedlings [21]. Furthermore, research has validated that Spd facilitates the binding of the cucumber GT-3b transcription factor to the s-adenosylmethionine synthase gene (*CsSAMs*), thus augmenting the expression of adenosylmethionine (SAM). SAM, being a crucial precursor for the synthesis of endogenous polyamines (PAs) and ethylene, plays a significant role in elevating the salt tolerance of cucumber seedlings [141]. In conclusion, Spd exhibits immense potential as an agent for improving salt tolerance in Cucurbitaceae plants through its multifaceted involvement in physiological and biochemical processes. Further investigation is warranted to elucidate its underlying mechanisms and explore its agricultural applications.

### 4.4. Signaling Molecules

Some signaling molecules, such as H_2_O_2_ [142], H_2_S [52], and NO [121,143], have also been found to contribute to ameliorating Cucurbitaceae salt stress. Under stress conditions, endogenous H_2_O_2_ often plays a curial role in reactive oxygen species scavenging [21]. Exogenous H_2_O_2_ can also be involved in the regulation of salt stress in plants [139]. It can mitigate the inhibitory effect of salt stress on cucumber seed germination by mediating the antioxidant enzyme system, ABA and GA, and can also mediate the antioxidant enzyme system to alleviate membrane lipid peroxidation, so as to enhance the resistance of melon seedlings to salt stress [144]. At present, some studies have found that H_2_S promotes the development of adventitious roots in cucumber under salt stress by regulating the content of osmotic substances in explants and improving antioxidant potential [59], and certain studies have demonstrated that H_2_S alleviates cucumber salt stress by preserving Na^+^/K^+^ homeostasis, regulating H_2_S metabolism and oxidative stress response [52]. Similar to H_2_S, exogenous NO attenuates mitochondrial oxidative stress induced by salt stress by increasing antioxidant enzyme activity [130]. In melon, exogenous NO treatment was effective in alleviating damage under salt stress, which was manifested in improving the transverse and longitudinal stems, seed cavities, pulp thickness, and yield of melon [130]. This is inseparable from the fact that exogenous NO can improve the growth, root activity, and antioxidant enzyme activity of melon seedlings under salt stress.

### 4.5. Plant Hormones

Some scientists have also explored the mechanism by which melatonin improves salt tolerance in plants [116]. Previous research has demonstrated that exogenous melatonin attenuates the suppressive effect of NaCl stress on cucumber seed germination primarily through regulating the biosynthesis and degradation of ABA and GA [145]. At the seedling stage, exogenous melatonin enhanced the salt tolerance of cucumber and bitter gourd by modulating ion balance, antioxidant systems, and genes related to the secondary metabolism [146]. ABA pretreatment can significantly enhance the tolerance of salt stress in rice and *Arabidopsis thaliana*, but it is not used in Cucurbitaceae [113]. Exogenous 2,4-epibrassinolide (EBR) is a homolog of the plant hormone BRs and is widely used in agricultural practices. The application of exogenous EBR enhanced salt tolerance by influencing cucumber seedling growth, photosynthetic pigments, the antioxidant defense system, ion homeostasis, the MAPK cascade reaction, and key genes involved in the SOS signaling pathway under salt stress [65]. Furthermore, to validate the hypothesis that the use of phytohormones could be a promising approach to boost tolerance, Parihar et al. subjected luffa seedlings to a combined NaCl and UV-B treatment, resulting in more severe growth impairment. However, the exogenous supplementation of methyl jasmonate (MeJA) or cis-(+)-12-oxo-phytodienoic acid (OPDA) was able to improve the seedlings’ growth performance by promoting nitrogen metabolism and photosynthesis, with the effects being more pronounced with OPDA [61]. However, whether OPDA can improve the salt tolerance of Cucurbitaceae still needs to be further experimentally explored and verified.

**Table 2:** Measures to improve the salt tolerance of Cucurbitaceae plants.

Measure	Species	Example	Principle	References
Nanoparticles	*Cucumis sativus*	CeO_2_	Modulates the antioxidant system to improve the salt tolerance of cucumber seedlings	[131]
Nanoparticles	*Cucumis sativus*	Silicon	Silicon increased the water conductivity of the root system and improved the water balance of seedlings; silicon reduces the Na^+^ content and reduces ionic toxicity	[128,132]
Nanoparticles	*Cucumis sativus*	PNC	Improves K^+^ absorption capacity and better maintains K^+^/Na^+^ ratio	[53]
Nanoparticles	*Cucumis sativus*	Se, SeO_2_, Mn_3_O_4_, etc.	Regulates ion channels and transporter-related genes to maintain ion homeostasis; directly or indirectly promotes reactive oxygen species scavenging mechanisms	[147]
Nanoparticles	*Momordica charantia*	Se-CS NPs	Induces multiple defense systems to alleviate salt stress	[133]
Graft	*Cucumis sativus*	Cucumber grafted onto pumpkin	Enhances Na^+^ efflux and induces stomatal closure	[135,136]
Graft	*Cucumis sativus*	Cucumber grafted onto luffa	Rootstock reduces Na^+^ transport to the shoot, facilitating Na^+^/K^+^ balance	[3]
Graft	*Cucumis melo*	Melon grafted onto luffa	Reduces the Na^+^ content of leaves and reduces salt toxicity	[136]
Graft	*Citrullus lanatus*	Watermelon grafted onto bottle gourd	Accumulation of less Na^+^ and improved ROS scavenging capacity	[135]
Exogenous polyamines	*Cucumis sativus*	Spd	Involved in cellular autophagy, degrades damaged proteins	[21]
Exogenous polyamines	*Cucumis sativus*	Spd	Enhances the expression of SAM for the synthesis of ethylene and PA	[13]
Signal molecules	*Cucumis sativus*	H_2_O_2_	Mediates the antioxidant enzyme system, ABA, and GA	[144]
Signal molecules	*Cucumis melo*	H_2_O_2_	Alleviates membrane lipid peroxidation to a certain extent, activates antioxidant enzyme activity in melon under stress	[148]
Signal molecules	*Cucumis sativus*	H_2_S	Enhances antioxidant capacity; improves Na^+^/K^+^ balance	[149]
Signal molecules	*Cucumis melo*	NO	Improves the growth, root activity, and antioxidant enzyme activity of melon seedlings under salt stress	[130]
Exogenous hormones	*Cucumis sativus*	Melatonin	Promotes the expression of endogenous hormones; improves photosynthesis, ion homeostasis, and activates a series of downstream signals	[145]
Exogenous hormones	*Momordica charantia*	Melatonin	Regulates ionic balance, antioxidant system and secondary metabolism-related genes	[146]
Exogenous hormones	*Cucumis sativus*	EBR	Enhances antioxidant capacity, maintain ionic homeostasis, and activates salt tolerance-related signaling pathways	[65]
Exogenous hormones	*Luffa cylindrica*	MEJA	Improves photosynthetic activity and nitrogen metabolism	[61]
Plant growth-promoting rhizobacteria	/	PGPR	Mediates the ethylene pathway and ROS scavenging	[150]

## 5. Conclusions and Prospects

The succulent flesh and delicious taste of cucurbit plants are widely cherished. In particular, cucumber, melon, and watermelon are common cucurbitaceous fruits and hold significant agronomic importance [151]. However, our comprehension of the molecular mechanisms of salt tolerance in cucurbit plants is far less advanced than that of *Arabidopsis* [97], rice [152], sorghum [2], maize [153], and other species. In particular, more detailed molecular regulation mechanisms and protein interactions and other fields are still very lacking. Therefore, we still have much progress to make in understanding salt stress in Cucurbitaceae.

While the increasingly refined reference genomes of cucurbit plants and the continuous development of modern molecular biology tools have made it possible to study the salt response mechanisms of these species, challenges such as time-consuming genetic transformation systems and low transformation rates mean that the molecular mechanisms of salt tolerance, such as in cucumber, are continuously lacking and primarily understood at the physiological and biochemical levels. Therefore, promoting the development of an effective genetic transformation system is a crucial avenue for exploring the molecular mechanisms of salt tolerance in cucurbit crops. Indeed, based on past research, the plant’s response to salt tolerance initially occurs in the roots, and Cucurbitaceae is particularly suited for grafting. Therefore, the use of grafting to focus on Cucurbitaceae root-stem-leaf salt signal transduction will be an interesting angle. Additionally, with the further improvement in genome assembly and the use of resequencing and other means [154,155], we might be able to analyze the different salt stress mechanisms between thick-skinned and thin-skinned melons from an evolutionary perspective. In addition, grafting and the application of exogenous nanomaterials and hormones can be used as an approach to alleviate salt stress in Cucurbitaceae. Nevertheless, the application rate of the donor and the selection of rootstock require further investigation tailored to specific plant species and growth stages.

Salt stress is a vital question to be addressed in the breeding of economic crops for resistance, which severely inhibits plant growth and development, influencing yield and quality. To tackle the issue of plant salt stress, future efforts should focus on two directions: Firstly, actively utilizing whole-genome analysis and transcriptome sequencing technologies to mine salt-tolerant genes from germplasm resources, exploring practical research on applying gene editing, agrobacterium infection, virus-induced gene silencing, and nanoparticle technologies for the functional validation of salt tolerance genes in cucurbit crops. This will enrich our knowledge on the salt stress regulatory network, clarify the mechanisms underlying salt response, and deepen our comprehension of the mechanisms underlying salt tolerance in cucurbit plants, strengthening the theoretical foundation to support the selection of salt-tolerant quality germplasm in cucurbit plants. Secondly, from a practical application standpoint, enhancing the salt tolerance of plants can be achieved through various biotechnological methods such as transgenic engineering, as well as through soil improvement and irrigation adjustments.

## Figures and Tables

**Table 1:** Cucumber mainly identified salt response genes and mechanisms.

Gene Name	Specific Mechanism	Key Regulatory Classification	References
*Cs* *A* *KT* *1*	Involved in K^+^ uptake under salt stress	Ionic homeostasis	[53]
*CsHAK* *5;3*	Involved in K^+^ uptake under salt stress	Ionic homeostasis	[54]
*CsSOS1/2/3*	Facilitates Na^+^ extrusion, regulates intracellular Na^+^/H^+^ homeostasis	Ionic homeostasis	[73]
*CsAQPs*	Involved in water molecule transport	Ionic homeostasis	[67]
*CsABF*	ABA-responsive element, involved in salt stress	Hormone regulation	[7]
*CsERF*	Ethylene response element, regulates salt stress through ethylene	Hormone regulation	[74]
*CsYUC*	Upregulated to elevate the auxin level	Hormone regulation	[75]
*CsCAT* *3*	Degrades H_2_O_2_ into H_2_O and O_2_, scavenges reactive oxygen species	ROS regulation	[76]
*Cs* *TGase*	Increases endogenous PA content and ROS scavenger capacity	ROS regulation	[77]
*CsMAPK* *s*	Involved in the signaling pathway of salt stress response, as well as the response to plant hormones	Signal transduction	[15]
*CsPP2* *-* *A1*	Osmoregulation and reactive oxygen species (ROS) homeostasis	Multiple regulation	[78]
*CsATG*	Autophagy gene, degrades proteins or organelles damaged by salt stress	Autophagy	[21]
*CsSAM*	Activates PA metabolic pathway, increases PA quantity	Signal transduction	[13]
*CsPNG1*	Involved in the ER-associated degradation pathway (ERAD)	Unknown	[79]
*CscircRNAs*	Potentially mediates transcription, signal transduction, cell cycle, metabolic adaptation, and ion homeostasis in salt stress response	Multiple regulation	[72]
*CsPti1-L*	A gene for a cytoplasmic protein kinase that may be involved in ABA signaling	Signal transduction	[80]
*CsbHLH041*	Enhances salt and ABA tolerance in cucumber seedlings	Transcriptional regulation	[81]
*CsNAC032*	The upregulation of salt stress may play a defensive role	Transcriptional regulation	[82]
*CsMYBs*	Involved in hormone signaling	Transcriptional regulation	[81]
*CsWRKY27, 41, 50*	It may be involved in ABA signaling pathway and Ros scavenging pathway	Transcriptional regulation	[83]
*CsCDPK6*	A gene for a membrane protein that is highly expressed under salt stress	Signal transduction	[84]
*CsPAO2*	Genes for key enzymes in polyamine metabolism	Signal transduction	[85]
*CsPAS3*	A gene that interacts with the PAO2 protein and is involved in polyamine transformation	ROS regulation	[85]
*CsSHMT3*	Serine hydroxymethyl transferase gene	Multiple regulation	[86]
*CsRBOHs*	Genes for the enzyme NADPH oxidase	ROS regulation	[87]
*CsZFPs*	May be involved in the regulation of plant hormones and/or abiotic stress responses	Transcriptional regulation	[88]
*CsMAX2*	A key gene of the strigolactones signal transduction pathway to improve stress tolerance	Hormone regulation	[89]
*CsPLD_α_*	A second messenger PA is produced to participate in the salt reaction	Signal transduction	[90]
*CsRAV1*	RAV transcription factor gene, an important regulator of salt response	Transcriptional regulation	[91]
*CsGPA1*	Inhibited the expression of *CsAQPs* in roots and leaves and reduced water content	Osmotic regulation	[67]
*CsSAMDC3*	Transcription regulates antioxidant enzyme activity	ROS regulation	[92]
*CsBPC2*	Transcription regulates ABA biosynthesis and expression of genes associated with ABA signaling	Hormone regulation	[93]
*CsTLP8*	Affects antioxidant enzyme activity and negatively regulates it, which may be related to ABA	ROS regulation	[94]

## References

[1] van Zelm E., Zhang Y., Testerink C. (2020). Salt Tolerance Mechanisms of Plants. Annu. Rev. Plant Biol..

[2] Zhang H., Yu F., Xie P., Sun S., Qiao X., Tang S., Chen C., Yang S., Mei C., Yang D. (2023). A Gγ Protein Regulates Alkaline Sensitivity in Crops. Science.

[3] Guo Z., Qin Y., Lv J., Wang X., Dong H., Dong X., Zhang T., Du N., Piao F. (2023). Luffa Rootstock Enhances Salt Tolerance and Improves Yield and Quality of Grafted Cucumber Plants by Reducing Sodium Transport to the Shoot. Environ. Pollut..

[4] Tang X., Mu X., Shao H., Wang H., Brestic M. (2015). Global Plant-Responding Mechanisms to Salt Stress: Physiological and Molecular Levels and Implications in Biotechnology. Crit. Rev. Biotechnol..

[5] Shelake R.M., Kadam U.S., Kumar R., Pramanik D., Singh A.K., Kim J.Y. (2022). Engineering Drought and Salinity Tolerance Traits in Crops through CRISPR-Mediated Genome Editing: Targets, Tools, Challenges, and Perspectives. Plant Commun..

[6] Zhu Y., Yuan G., Gao B., An G., Li W., Si W., Sun D., Liu J. (2022). Comparative Transcriptome Profiling Provides Insights into Plant Salt Tolerance in Watermelon (*Citrullus lanatus*). Life.

[7] Niu M., Sun S., Nawaz M.A., Sun J., Cao H., Lu J., Huang Y., Bie Z. (2019). Grafting Cucumber Onto Pumpkin Induced Early Stomatal Closure by Increasing ABA Sensitivity under Salinity Conditions. Front. Plant Sci..

[8] Qin H., Wang J., Chen X., Wang F., Peng P., Zhou Y., Miao Y., Zhang Y., Gao Y., Qi Y. (2019). Rice OsDOF15 Contributes to Ethylene-Inhibited Primary Root Elongation under Salt Stress. New Phytol..

[9] Seleiman M.F., Semida W.M., Rady M.M., Mohamed G.F., Hemida K.A., Alhammad B.A., Hassan M.M., Shami A. (2020). Sequential Application of Antioxidants Rectifies Ion Imbalance and Strengthens Antioxidant Systems in Salt-Stressed Cucumber. Plants.

[10] Yao H.Y., Xue H.W. (2018). Phosphatidic Acid Plays Key Roles Regulating Plant Development and Stress Responses. J. Integr. Plant Biol..

[11] Ji H., Pardo J.M., Batelli G., Van Oosten M.J., Bressan R.A., Li X. (2013). The Salt Overly Sensitive (SOS) Pathway: Established and Emerging Roles. Mol. Plant.

[12] Huang S., Hu L., Zhang S., Zhang M., Jiang W., Wu T., Du X. (2021). Rice OsWRKY50 Mediates ABA-Dependent Seed Germination and Seedling Growth, and ABA-Independent Salt Stress Tolerance. Int. J. Mol. Sci..

[13] Wang Y., Gong X., Liu W., Kong L., Si X., Guo S., Sun J. (2020). Gibberellin Mediates Spermidine-Induced Salt Tolerance and the Expression of GT-3b in Cucumber. Plant Physiol. Biochem..

[14] Castro B., Citterico M., Kimura S., Stevens D.M., Wrzaczek M., Coaker G. (2021). Stress-Induced Reactive Oxygen Species Compartmentalization, Perception and Signalling. Nat. Plants.

[15] Wang J., Pan C., Wang Y., Ye L., Wu J., Chen L., Zou T., Lu G. (2015). Genome-Wide Identification of MAPK, MAPKK, and MAPKKK Gene Families and Transcriptional Profiling Analysis during Development and Stress Response in Cucumber. BMC Genom..

[16] Fang L., Wei X.Y., Liu L.Z., Zhou L.X., Tian Y.P., Geng C., Li X.D. (2021). A Tobacco Ringspot Virus-Based Vector System for Gene and microRNA Function Studies in Cucurbits. Plant Physiol..

[17] Fu H., Yu X., Jiang Y., Wang Y., Yang Y., Chen S., Chen Q., Guo Y. (2023). SALT OVERLY SENSITIVE 1 Is Inhibited by Clade D Protein Phosphatase 2C D6 and D7 in *Arabidopsis thaliana*. Plant Cell.

[18] Cao Y., Zhou X., Song H., Zhang M., Jiang C. (2023). Advances in Deciphering Salt Tolerance Mechanism in Maize. Crop J..

[19] Behera T.K., Krishna R., Ansari W.A., Aamir M., Kumar P., Kashyap S.P., Pandey S., Kole C. (2021). Approaches Involved in the Vegetable Crops Salt Stress Tolerance Improvement: Present Status and Way Ahead. Front. Plant Sci..

[20] Yang Y., Ahammed G.J., Wan C., Liu H., Chen R., Zhou Y. (2019). Comprehensive Analysis of TIFY Transcription Factors and Their Expression Profiles under Jasmonic Acid and Abiotic Stresses in Watermelon. Int. J. Genom..

[21] Zhang Y., Wang Y., Wen W., Shi Z., Gu Q., Ahammed G.J., Cao K., Shah Jahan M., Shu S., Wang J. (2021). Hydrogen Peroxide Mediates Spermidine-Induced Autophagy to Alleviate Salt Stress in Cucumber. Autophagy.

[22] Mishra P., Mishra J., Arora N.K. (2021). Plant Growth Promoting Bacteria for Combating Salinity Stress in Plants-Recent Developments and Prospects: A Review. Microbiol. Res..

[23] Yang L.I., Kai L., Jipeng W., Lan Z., Xin L.I., Wenyan H., Qingyun L.I., University H.A. (2018). Effects of Various Concentrations of EGCG on Seed Germination and Resistance in Cucumber under NaCl Stress. Acta Agric. Zhejiangensis.

[24] Li L., Du L., Cao Q., Yang Z., Liu Y., Yang H., Duan X., Meng Z. (2023). Salt Tolerance Evaluation of Cucumber Germplasm under Sodium Chloride Stress. Plants.

[25] Chevilly S., Dolz-Edo L., Martínez-Sánchez G., Morcillo L., Vilagrosa A., López-Nicolás J.M., Blanca J., Yenush L., Mulet J.M. (2021). Distinctive Traits for Drought and Salt Stress Tolerance in Melon (*Cucumis melo* L.). Front. Plant Sci..

[26] Marium A., Kausar A., Ali Shah S.M., Ashraf M.Y., Akhtar N., Akram M., Riaz M. (2019). Assessment of Cucumber Genotypes for Salt Tolerance Based on Germination and Physiological Indices. Dose Response.

[27] Yasar F., Kusvuran S., Ellialtioglu S. (2006). Determination of Anti-Oxidant Activities in Some Melon (*Cucumis melo* L.) Varieties and Cultivars under Salt Stress. J. Hortic. Sci. Biotechnol..

[28] Liu D., Dong S., Miao H., Liu X., Li C., Han J., Zhang S., Gu X. (2022). A Large-Scale Genomic Association Analysis Identifies the Candidate Genes Regulating Salt Tolerance in Cucumber (*Cucumis sativus* L.) Seedlings. Int. J. Mol. Sci..

[29] Liu D., Dong S., Bo K., Miao H., Li C., Zhang Y., Zhang S., Gu X. (2021). Identification of QTLs Controlling Salt Tolerance in Cucumber (*Cucumis sativus* L.) Seedlings. Plants.

[30] Sarabi B., Bolandnazar S., Ghaderi N., Ghashghaie J. (2017). Genotypic Differences in Physiological and Biochemical Responses to Salinity Stress in Melon (*Cucumis melo* L.) Plants: Prospects for Selection of Salt Tolerant Landraces. Plant Physiol. Biochem..

[31] Tarchoun N., Saadaoui W., Mezghani N., Pavli O.I., Falleh H., Petropoulos S.A. (2022). The Effects of Salt Stress on Germination, Seedling Growth and Biochemical Responses of Tunisian Squash (*Cucurbita maxima* Duchesne) Germplasm. Plants.

[32] Abdel-Farid I.B., Marghany M.R., Rowezek M.M., Sheded M.G. (2020). Effect of Salinity Stress on Growth and Metabolomic Profiling of *Cucumis sativus* and *Solanum lycopersicum*. Plants.

[33] Liu Y., Zhang W., Elango D., Liu H., Jin D., Wang X., Wu Y. (2023). Metabolome and Transcriptome Analysis Reveals Molecular Mechanisms of Watermelon under Salt Stress. Environ. Exp. Bot..

[34] Yang Y., Wang L., Tian J., Li J., Sun J., He L., Guo S., Tezuka T. (2012). Proteomic Study Participating the Enhancement of Growth and Salt Tolerance of Bottle Gourd Rootstock-Grafted Watermelon Seedlings. Plant Physiol. Biochem..

[35] Ondrasek G., Romic D., Rengel Z., Romic M., Zovko M. (2009). Cadmium Accumulation by Muskmelon under Salt Stress in Contaminated Organic Soil. Sci. Total Environ..

[36] Akrami M., Arzani A. (2019). Inheritance of Fruit Yield and Quality in Melon (*Cucumis melo* L.) Grown under Field Salinity Stress. Sci. Rep..

[37] Sanoubar R., Orsini F., Gianquinto G. (2013). Ionic Partitioning and Stomatal Regulation: Dissecting Functional Elements of the Genotypic Basis of Salt Stress Adaptation in Grafted Melon. Plant Signal. Behav..

[38] Akrami M., Arzani A., Majnoun Z. (2019). Leaf Ion Content, Yield and Fruit Quality of Field-Grown Melon under Saline Conditions. Ex. Agric..

[39] Wei S., Wang L., Zhang Y., Huang D. (2013). Identification of Early Response Genes to Salt Stress in Roots of Melon (*Cucumis melo* L.) Seedlings. Mol. Biol. Rep..

[40] Sohail H., Noor I., Nawaz M., Ma M., Shireen F., Huang Y., Yang L., Bie Z. (2022). Genome-Wide Identification of Plasma-Membrane Intrinsic Proteins in Pumpkin and Functional Characterization of CmoPIP1-4 under Salinity Stress. Environ. Exp. Bot..

[41] Niu M., Huang Y., Sun S., Sun J., Cao H., Shabala S., Bie Z. (2018). Root Respiratory Burst Oxidase Homologue-Dependent H_2_O_2_ Production Confers Salt Tolerance on a Grafted Cucumber by Controlling Na^+^ Exclusion and Stomatal Closure. J. Exp. Bot..

[42] Rouphael Y., Cardarelli M., Rea E., Colla G. (2012). Improving Melon and Cucumber Photosynthetic Activity, Mineral Composition, and Growth Performance under Salinity Stress by Grafting onto Cucurbita Hybrid Rootstocks. Photosynthetica.

[43] Huang Y., Cao H., Yang L., Chen C., Shabala L., Xiong M., Niu M., Liu J., Zheng Z., Zhou L. (2019). Tissue-Specific Respiratory Burst Oxidase Homolog-Dependent H_2_O_2_ Signaling to the Plasma Membrane H^+^-ATPase Confers Potassium Uptake and Salinity Tolerance in Cucurbitaceae. J. Exp. Bot..

[44] Fu A., Zheng Y., Guo J., Grierson D., Zhao X., Wen C., Liu Y., Li J., Zhang X., Yu Y. (2022). Telomere-to-Telomere Genome Assembly of Bitter Melon (*Momordica charantia* L. Var. Abbreviata Ser.) Reveals Fruit Development, Composition and Ripening Genetic Characteristics. Hortic. Res..

[45] Cebrián G., Iglesias-Moya J., García A., Martínez J., Romero J., Regalado J.J., Martínez C., Valenzuela J.L., Jamilena M. (2021). Involvement of Ethylene Receptors in the Salt Tolerance Response of *Cucurbita pepo*. Hortic. Res..

[46] Ma L., Ye J., Yang Y., Lin H., Yue L., Luo J., Long Y., Fu H., Liu X., Zhang Y. (2019). The SOS2-SCaBP8 Complex Generates and Fine-Tunes an AtANN4-Dependent Calcium Signature under Salt Stress. Dev. Cell.

[47] Steinhorst L., He G., Moore L.K., Schültke S., Schmitz-Thom I., Cao Y., Hashimoto K., Andrés Z., Piepenburg K., Ragel P. (2022). A Ca^2+^-Sensor Switch for Tolerance to Elevated Salt Stress in Arabidopsis. Dev. Cell.

[48] Chen C., He G., Li J., Perez-Hormaeche J., Becker T., Luo M., Wallrad L., Gao J., Li J., Pardo J.M. (2023). A Salt Stress-Activated GSO1-SOS2-SOS1 Module Protects the Arabidopsis Root Stem Cell Niche by Enhancing Sodium Ion Extrusion. EMBO J..

[49] Flores-León A., García-Martínez S., González V., Garcés-Claver A., Martí R., Julián C., Sifres A., Pérez-de-Castro A., Díez M.J., López C. (2021). Grafting Snake Melon [*Cucumis melo* L. Subsp. Melo Var. *Flexuosus* (L.) Naudin] in Organic Farming: Effects on Agronomic Performance; Resistance to Pathogens; Sugar, Acid, and VOC Profiles; and Consumer Acceptance. Front. Plant Sci..

[50] Akrami M., Arzani A. (2018). Physiological Alterations Due to Field Salinity Stress in Melon (*Cucumis melo* L.). Acta Physiol. Plant..

[51] Qian Z.J., Song J.J., Chaumont F., Ye Q. (2015). Differential Responses of Plasma Membrane Aquaporins in Mediating Water Transport of Cucumber Seedlings under Osmotic and Salt Stresses. Plant Cell Environ..

[52] Jiang J.L., Tian Y., Li L., Yu M., Hou R.-P., Ren X.M. (2019). H2S Alleviates Salinity Stress in Cucumber by Maintaining the Na^+^/K^+^ Balance and Regulating H_2_S Metabolism and Oxidative Stress Response. Front. Plant Sci..

[53] Peng Y., Chen L., Zhu L., Cui L., Yang L., Wu H., Bie Z. (2022). CsAKT1 Is a Key Gene for the CeO_2_ Nanoparticle’s Improved Cucumber Salt Tolerance: A Validation from CRISPR-Cas9 Lines. Environ. Sci. Nano.

[54] Peng Y., Cao H., Peng Z., Zhou L., Sohail H., Cui L., Yang L., Huang Y., Bie Z. (2023). Transcriptomic and Functional Characterization Reveals CsHAK5;3 as a Key Player in K^+^ Homeostasis in Grafted Cucumbers under Saline Conditions. Plant Sci..

[55] Gao L.W., Yang S.L., Wei S.W., Huang D.F., Zhang Y.D. (2020). Supportive Role of the Na^+^ Transporter CmHKT1;1 from *Cucumis melo* in Transgenic Arabidopsis Salt Tolerance through Improved K^+^/Na^+^ Balance. Plant Mol. Biol..

[56] Wei L., Liu L., Chen Z., Huang Y., Yang L., Wang P., Xue S., Bie Z. (2023). CmCNIH1 Improves Salt Tolerance by Influencing the Trafficking of CmHKT1;1 in Pumpkin. Plant J..

[57] Zhang Y.D., Véry A.A., Wang L.M., Deng Y.W., Sentenac H., Huang D.F. (2011). A K^+^ Channel from Salt-Tolerant Melon Inhibited by Na^+^. New Phytol..

[58] Yu J., Yu J., Liao W., Xie J., Wu Y. (2020). Ethylene Was Involved in Ca^2+^-Regulated Na^+^ Homeostasis, Na^+^ Transport and Cell Ultrastructure During Adventitious Rooting in Cucumber Explants under Salt Stress. J. Plant Biol..

[59] Liu Y., Wei L., Feng L., Zhang M., Hu D., Tie J., Liao W. (2022). Hydrogen Sulfide Promotes Adventitious Root Development in Cucumber under Salt Stress by Enhancing Antioxidant Ability. Plants.

[60] Zhang G., Ding Q., Wei B. (2021). Genome-Wide Identification of Superoxide Dismutase Gene Families and Their Expression Patterns under Low-Temperature, Salt and Osmotic Stresses in Watermelon and Melon. 3 Biotech.

[61] Parihar P., Singh R., Singh A., Prasad S.M. (2021). Role of Oxylipin on Luffa Seedlings Exposed to NaCl and UV-B Stresses: An Insight into Mechanism. Plant Physiol. Biochem..

[62] Li H., Chang J., Chen H., Wang Z., Gu X., Wei C., Zhang Y., Ma J., Yang J., Zhang X. (2017). Exogenous Melatonin Confers Salt Stress Tolerance to Watermelon by Improving Photosynthesis and Redox Homeostasis. Front. Plant Sci..

[63] Shu S., Guo S.-R., Sun J., Yuan L.Y. (2012). Effects of Salt Stress on the Structure and Function of the Photosynthetic Apparatus in *Cucumis Sativus* and Its Protection by Exogenous Putrescine. Physiol. Plant.

[64] Gurmani A.R., Khan S.U., Ali A., Rubab T., Schwinghamer T., Jilani G., Farid A., Zhang J. (2018). Salicylic Acid and Kinetin Mediated Stimulation of Salt Tolerance in Cucumber (*Cucumis sativus* L.) Genotypes Varying in Salinity Tolerance. Hortic. Environ. Biotechnol..

[65] He X., Wan Z., Jin N., Jin L., Zhang G., Lyu J., Liu Z., Luo S., Yu J. (2022). Enhancement of Cucumber Resistance under Salt Stress by 2, 4-Epibrassinolide Lactones. Front. Plant Sci..

[66] Chen J.B., Zhang F.R., Huang D.F., Zhang L.D., Zhang Y.D. (2014). Transcriptome Analysis of Transcription Factors in Two Melon (*Cucumis melo* L.) Cultivars under Salt Stress. Plant Physiol. J..

[67] Yan Y., Sun M., Li Y., Wang J., He C., Yu X. (2020). The CsGPA1-CsAQPs Module Is Essential for Salt Tolerance of Cucumber Seedlings. Plant Cell Rep..

[68] Li J., Yang C., Xu J., Lu H., Liu J. (2023). The Hot Science in Rice Research: How Rice Plants Cope with Heat Stress. Plant Cell Environ..

[69] Yin J., Liu Y., Lu L., Zhang J., Chen S., Wang B. (2022). Comparison of Tolerant and Susceptible Cultivars Revealed the Roles of Circular RNAs in Rice Responding to Salt Stress. Plant Growth Regul..

[70] Wang Z., Li N., Yu Q., Wang H. (2021). Genome-Wide Characterization of Salt-Responsive miRNAs, circRNAs and Associated ceRNA Networks in Tomatoes. Int. J. Mol. Sci..

[71] Liu P., Zhu Y., Liu H., Liang Z., Zhang M., Zou C., Yuan G., Gao S., Pan G., Shen Y. (2022). A Combination of a Genome-Wide Association Study and a Transcriptome Analysis Reveals circRNAs as New Regulators Involved in the Response to Salt Stress in Maize. Int. J. Mol. Sci..

[72] Zhu Y.X., Jia J.H., Yang L., Xia Y.C., Zhang H.L., Jia J.B., Zhou R., Nie P.Y., Yin J.L., Ma D.F. (2019). Identification of Cucumber Circular RNAs Responsive to Salt Stress. BMC Plant Biol..

[73] Bin L.I., Guo S.R., Sun J., Juan L.I. (2011). Effects of Exogenous Spermidine on Free Polyamine Content and Polyamine Biosynthesis Gene Expression in Cucumber Seedlings under Salt Stress. Chin. J. Eco-Agric..

[74] Reda M., Golicka A., Kabała K., Janicka M. (2018). Involvement of NR and PM-NR in NO Biosynthesis in Cucumber Plants Subjected to Salt Stress. Plant Sci..

[75] Yan S., Che G., Ding L., Chen Z., Liu X., Wang H., Zhao W., Ning K., Zhao J., Tesfamichael K. (2016). Different Cucumber CsYUC Genes Regulate Response to Abiotic Stresses and Flower Development. Sci. Rep..

[76] Zijian Y., Yong Z., Lingli G., Guanghua L., Qiang L., Yaping X., Lunwei J., Yingui Y., Agriculture S.O., University J.A. (2018). Expression of Cucumber CsCAT3 Gene under Stress and Its Salt Tolerance in Transgenic Arabidopsis Thaliana. Mol. Plant Breed..

[77] Shu S., Tang Y., Zhou X., Jahan M.S., Sun J., Wang Y., Guo S. (2020). Physiological Mechanism of Transglutaminase-Mediated Improvement in Salt Tolerance of Cucumber Seedlings. Plant Physiol. Biochem..

[78] Si Y., Fan H., Lu H., Li Y., Guo Y., Liu C., Chai L., Du C. (2023). *Cucumis sativus* PHLOEM PROTEIN 2-A1 like Gene Positively Regulates Salt Stress Tolerance in Cucumber Seedlings. Plant Mol. Biol..

[79] Hou K., Wang Y., Tao M.Q., Jahan M.S., Shu S., Sun J., Guo S.R. (2020). Characterization of the CsPNG1 Gene from Cucumber and Its Function in Response to Salinity Stress. Plant Physiol. Biochem..

[80] Oh S.K., Jang H.A., Lee S.S., Cho H.S., Lee D.H., Choi D., Kwon S.Y. (2014). Cucumber Pti1-L Is a Cytoplasmic Protein Kinase Involved in Defense Responses and Salt Tolerance. J. Plant Physiol..

[81] Li J., Wang T., Han J., Ren Z. (2020). Genome-Wide Identification and Characterization of Cucumber bHLH Family Genes and the Functional Characterization of CsbHLH041 in NaCl and ABA Tolerance in Arabidopsis and Cucumber. BMC Plant Biol..

[82] Zhang X.M., Yu H.J., Sun C., Deng J., Zhang X., Liu P., Li Y.Y., Li Q., Jiang W.J. (2017). Genome-Wide Characterization and Expression Profiling of the NAC Genes under Abiotic Stresses in *Cucumis sativus*. Plant Physiol. Biochem..

[83] Chen C., Chen X., Han J., Lu W., Ren Z. (2020). Genome-Wide Analysis of the WRKY Gene Family in the Cucumber Genome and Transcriptome-Wide Identification of WRKY Transcription Factors That Respond to Biotic and Abiotic Stresses. BMC Plant Biol..

[84] Zhu H., He M., Jahan M., Wu J., Gu Q., Shu S., Sun J., Guo S. (2021). CsCDPK6, a CsSAMS1-Interacting Protein, Affects Polyamine/Ethylene Biosynthesis in Cucumber and Enhances Salt Tolerance by Overexpression in Tobacco. Int. J. Mol. Sci..

[85] Wu J., Zhu M., Liu W., Jahan M.S., Gu Q., Shu S., Sun J., Guo S. (2022). CsPAO2 Improves Salt Tolerance of Cucumber through the Interaction with CsPSA3 by Affecting Photosynthesis and Polyamine Conversion. Int. J. Mol. Sci..

[86] Zhang Z., Hou X., Gao R., Li Y., Ding Z., Huang Y., Yao K., Yao Y., Liang C., Liao W. (2024). CsSHMT3 Gene Enhances the Growth and Development in Cucumber Seedlings under Salt Stress. Plant Mol. Biol..

[87] Kabała K., Reda M., Wdowikowska A., Janicka M. (2022). Role of Plasma Membrane NADPH Oxidase in Response to Salt Stress in Cucumber Seedlings. Antioxidants.

[88] Yin J., Wang L., Zhao J., Li Y., Huang R., Jiang X., Zhou X., Zhu X., He Y., He Y. (2020). Genome-Wide Characterization of the C2H2 Zinc-Finger Genes in *Cucumis sativus* and Functional Analyses of Four CsZFPs in Response to Stresses. BMC Plant Biol..

[89] Zhang R., Dong Y., Li Y., Ren G., Chen C., Jin X. (2023). SLs Signal Transduction Gene CsMAX2 of Cucumber Positively Regulated to Salt, Drought and ABA Stress in *Arabidopsis Thaliana* L.. Gene.

[90] Li S., Huang M., Di Q., Ji T., Wang X., Wei M., Shi Q., Li Y., Gong B., Yang F. (2015). The Functions of a Cucumber Phospholipase D Alpha Gene (CsPLDα) in Growth and Tolerance to Hyperosmotic Stress. Plant Physiol. Biochem..

[91] Li J., Song C., Li H., Wang S., Hu L., Yin Y., Wang Z., He W. (2023). Comprehensive Analysis of Cucumber RAV Family Genes and Functional Characterization of CsRAV1 in Salt and ABA Tolerance in Cucumber. Front. Plant Sci..

[92] Zhu M., Chen G., Wu J., Wang J., Wang Y., Guo S., Shu S. (2023). Identification of Cucumber S-Adenosylmethionine Decarboxylase Genes and Functional Analysis of CsSAMDC3 in Salt Tolerance. Front. Plant Sci..

[93] Li S., Sun M., Miao L., Di Q., Lv L., Yu X., Yan Y., He C., Wang J., Shi A. (2023). Multifaceted Regulatory Functions of CsBPC2 in Cucumber under Salt Stress Conditions. Hortic. Res..

[94] Li S., Wang Z., Wang F., Lv H., Cao M., Zhang N., Li F., Wang H., Li X., Yuan X. (2021). A Tubby-like Protein CsTLP8 Acts in the ABA Signaling Pathway and Negatively Regulates Osmotic Stresses Tolerance during Seed Germination. BMC Plant Biol..

[95] Rus A., Lee B., Muñoz-Mayor A., Sharkhuu A., Miura K., Zhu J.K., Bressan R.A., Hasegawa P.M. (2004). AtHKT1 Facilitates Na^+^ Homeostasis and K^+^ Nutrition in Planta. Plant Physiol..

[96] Li J., Shen L., Han X., He G., Fan W., Li Y., Yang S., Zhang Z., Yang Y., Jin W. (2023). Phosphatidic Acid–Regulated SOS2 Controls Sodium and Potassium Homeostasis in Arabidopsis under Salt Stress. EMBO J..

[97] Lu K.K., Song R.F., Guo J.X., Zhang Y., Zuo J.X., Chen H.H., Liao C.Y., Hu X.Y., Ren F., Lu Y.T. (2023). CycC1;1–WRKY75 Complex-Mediated Transcriptional Regulation of SOS1 Controls Salt Stress Tolerance in Arabidopsis. Plant Cell.

[98] Hao R., Zhou W., Li J., Luo M., Scheres B., Guo Y. (2023). On Salt Stress, PLETHORA Signaling Maintains Root Meristems. Dev. Cell.

[99] Quan R., Lin H., Mendoza I., Zhang Y., Cao W., Yang Y., Shang M., Chen S., Pardo J.M., Guo Y. (2007). SCABP8/CBL10, a Putative Calcium Sensor, Interacts with the Protein Kinase SOS2 to Protect Arabidopsis Shoots from Salt Stress. Plant Cell.

[100] Yin X., Xia Y., Xie Q., Cao Y., Wang Z., Hao G., Song J., Zhou Y., Jiang X. (2020). The Protein Kinase Complex CBL10-CIPK8-SOS1 Functions in Arabidopsis to Regulate Salt Tolerance. J. Exp. Bot..

[101] Li B., Wang D., Han L., Zhang Y., Zhang L., Liu D. (2020). Effect of Polyamines on SOS2 Family Gene Expression in Cucumber under Salt Stress. Acta Bot. Boreal. Occident. Sin..

[102] Chen K., Li G.J., Bressan R.A., Song C.P., Zhu J.K., Zhao Y. (2020). Abscisic Acid Dynamics, Signaling, and Functions in Plants. J. Integr. Plant Biol..

[103] Yan X., Yue Z., Pan X., Si F., Li J., Chen X., Li X., Luan F., Yang J., Zhang X. (2022). The HD-ZIP Gene Family in Watermelon: Genome-Wide Identification and Expression Analysis under Abiotic Stresses. Genes.

[104] Zhou Y., Cheng Y., Wan C., Li J., Yang Y., Chen J. (2020). Genome-Wide Characterization and Expression Analysis of the Dof Gene Family Related to Abiotic Stress in Watermelon. PeerJ.

[105] Song Q., Joshi M., Joshi V. (2020). Transcriptomic Analysis of Short-Term Salt Stress Response in Watermelon Seedlings. Int. J. Mol. Sci..

[106] Du F., Wang Y., Wang J., Li Y., Zhang Y., Zhao X., Xu J., Li Z., Zhao T., Wang W. (2023). The bHLH Transcription Factor Gene, OsbHLH38, Plays a Key Role in Controlling Rice Salt Tolerance. J. Integr. Plant Biol..

[107] Peng Y., Cao H., Cui L., Wang Y., Wei L., Geng S., Yang L., Huang Y., Bie Z. (2023). CmoNAC1 in Pumpkin Rootstocks Improves Salt Tolerance of Grafted Cucumbers by Binding to the Promoters of CmoRBOHD1, CmoNCED6, CmoAKT1;2 and CmoHKT1;1 to Regulate H_2_O_2_, ABA Signaling and K^+^/Na^+^ Homeostasis. Hortic. Res..

[108] Liu H., Tang X., Zhang N., Li S., Si H. (2023). Role of bZIP Transcription Factors in Plant Salt Stress. Int. J. Mol. Sci..

[109] Wei S., Gao L., Zhang Y., Zhang F., Yang X., Huang D. (2016). Genome-Wide Investigation of the NAC Transcription Factor Family in Melon (*Cucumis melo* L.) and Their Expression Analysis under Salt Stress. Plant Cell Rep..

[110] Boonyaves K., Ang M.C.-Y., Park M., Cui J., Khong D.T., Singh G.P., Koman V.B., Gong X., Porter T.K., Choi S.W. (2023). Near-Infrared Fluorescent Carbon Nanotube Sensors for the Plant Hormone Family Gibberellins. Nano Lett..

[111] Ribba T., Garrido-Vargas F., O’Brien J.A. (2020). Auxin-Mediated Responses under Salt Stress: From Developmental Regulation to Biotechnological Applications. J. Exp. Bot..

[112] Huang X., Hou L., Meng J., You H., Li Z., Gong Z., Yang S., Shi Y. (2018). The Antagonistic Action of Abscisic Acid and Cytokinin Signaling Mediates Drought Stress Response in Arabidopsis. Mol. Plant.

[113] Habibpourmehraban F., Wu Y., Masoomi-Aladizgeh F., Amirkhani A., Atwell B.J., Haynes P.A. (2023). Pre-Treatment of Rice Plants with ABA Makes Them More Tolerant to Multiple Abiotic Stress. Int. J. Mol. Sci..

[114] Zhang N., Zhang H.J., Zhao B., Sun Q.Q., Cao Y.Y., Li R., Wu X.X., Weeda S., Li L., Ren S. (2014). The RNA-Seq Approach to Discriminate Gene Expression Profiles in Response to Melatonin on Cucumber Lateral Root Formation. J. Pineal Res..

[115] Chang J., Guo Y., Yan J., Zhang Z., Yuan L., Wei C., Zhang Y., Ma J., Yang J., Zhang X. (2021). The Role of Watermelon Caffeic Acid O-Methyltransferase (ClCOMT1) in Melatonin Biosynthesis and Abiotic Stress Tolerance. Hortic. Res..

[116] Zhan H., Nie X., Zhang T., Li S., Wang X., Du X., Tong W., Song W. (2019). Melatonin: A Small Molecule but Important for Salt Stress Tolerance in Plants. Int. J. Mol. Sci..

[117] Monihan S.M., Magness C.A., Yadegari R., Smith S.E., Schumaker K.S. (2016). Arabidopsis CALCINEURIN B-LIKE10 Functions Independently of the SOS Pathway during Reproductive Development in Saline Conditions. Plant Physiol..

[118] Yang Y., Zhang C., Tang R.J., Xu H.X., Lan W.Z., Zhao F., Luan S. (2019). Calcineurin B-Like Proteins CBL4 and CBL10 Mediate Two Independent Salt Tolerance Pathways in Arabidopsis. Int. J. Mol. Sci..

[119] Zhu J.K. (2001). Cell Signaling under Salt, Water and Cold Stresses. Curr. Opin. Plant Biol..

[120] Yang S., Xiong X., Arif S., Gao L., Zhao L., Shah I.H., Zhang Y. (2020). A Calmodulin-like CmCML13 from *Cucumis melo* Improved Transgenic Arabidopsis Salt Tolerance through Reduced Shoot’s Na^+^, and Also Improved Drought Resistance. Plant Physiol. Biochem..

[121] Sun Y., Ma C., Kang X., Zhang L., Wang J., Zheng S., Zhang T. (2021). Hydrogen Sulfide and Nitric Oxide Are Involved in Melatonin-Induced Salt Tolerance in Cucumber. Plant Physiol. Biochem..

[122] Shen L., Zhuang B., Wu Q., Zhang H., Nie J., Jing W., Yang L., Zhang W. (2019). Phosphatidic Acid Promotes the Activation and Plasma Membrane Localization of MKK7 and MKK9 in Response to Salt Stress. Plant Sci..

[123] Wang F., Jing W., Zhang W. (2014). The Mitogen-Activated Protein Kinase Cascade MKK1–MPK4 Mediates Salt Signaling in Rice. Plant Sci..

[124] He X., Wang C., Wang H., Li L., Wang C. (2020). The Function of MAPK Cascades in Response to Various Stresses in Horticultural Plants. Front. Plant Sci..

[125] Song Q., Li D., Dai Y., Liu S., Huang L., Hong Y., Zhang H., Song F. (2015). Characterization, Expression Patterns and Functional Analysis of the MAPK and MAPKK Genes in Watermelon (*Citrullus lanatus*). BMC Plant Biol..

[126] Zhang X., Li Y., Xing Q., Yue L., Qi H. (2020). Genome-Wide Identification of Mitogen-Activated Protein Kinase (MAPK) Cascade and Expression Profiling of CmMAPKs in Melon (*Cucumis melo* L.). PLoS ONE.

[127] Mastrochirico M., Spanò R., Mascia T. (2022). Grafting to Manage Infections of the Emerging Tomato Leaf Curl New Delhi Virus in Cucurbits. Plants.

[128] Yin J., Jia J., Lian Z., Hu Y., Guo J., Huo H., Zhu Y., Gong H. (2019). Silicon Enhances the Salt Tolerance of Cucumber through Increasing Polyamine Accumulation and Decreasing Oxidative Damage. Ecotoxicol. Environ. Saf..

[129] Shu S., Yuan L.Y., Guo S.R., Sun J., Yuan Y.H. (2013). Effects of Exogenous Spermine on Chlorophyll Fluorescence, Antioxidant System and Ultrastructure of Chloroplasts in *Cucumis sativus* L. under Salt Stress. Plant Physiol. Biochem..

[130] Shi Q., Ding F., Wang X., Wei M. (2007). Exogenous Nitric Oxide Protect Cucumber Roots against Oxidative Stress Induced by Salt Stress. Plant Physiol. Biochem..

[131] Chen L., Peng Y., Zhu L., Huang Y., Bie Z., Wu H. (2022). CeO_2_ Nanoparticles Improved Cucumber Salt Tolerance Is Associated with Its Induced Early Stimulation on Antioxidant System. Chemosphere.

[132] Wang S., Liu P., Chen D., Yin L., Li H., Deng X. (2015). Silicon Enhanced Salt Tolerance by Improving the Root Water Uptake and Decreasing the Ion Toxicity in Cucumber. Front. Plant Sci..

[133] Sheikhalipour M., Mohammadi S.A., Esmaielpour B., Spanos A., Mahmoudi R., Mahdavinia G.R., Milani M.H., Kahnamoei A., Nouraein M., Antoniou C. (2023). Seedling Nanopriming with Selenium-Chitosan Nanoparticles Mitigates the Adverse Effects of Salt Stress by Inducing Multiple Defence Pathways in Bitter Melon Plants. Int. J. Biol. Macromol..

[134] Lang X., Zhao X., Zhao J., Ren T., Nie L., Zhao W. (2024). MicroRNA Profiling Revealed the Mechanism of Enhanced Cold Resistance by Grafting in Melon (*Cucumis melo* L.). Plants.

[135] Bőhm V., Fekete D., Balázs G., Gáspár L., Kappel N. (2017). Salinity Tolerance of Grafted Watermelon Seedlings. Acta Biol. Hung..

[136] Xu Y., Guo S., Li H., Sun H., Lu N., Shu S., Sun J. (2017). Resistance of Cucumber Grafting Rootstock Pumpkin Cultivars to Chilling and Salinity Stresses. Hortic. Sci. Technol..

[137] Wang Y., Zhou J., Wen W., Sun S., Shu S., Guo S. (2023). Transcriptome and Proteome Analysis Identifies Salt Stress Response Genes in Bottle Gourd Rootstock-Grafted Watermelon Seedlings. Agronomy.

[138] Li B., He L., Guo S., Li J., Yang Y., Yan B., Sun J., Li J. (2013). Proteomics Reveal Cucumber Spd-Responses under Normal Condition and Salt Stress. Plant Physiol. Biochem..

[139] Wu J., Shu S., Li C., Sun J., Guo S. (2018). Spermidine-Mediated Hydrogen Peroxide Signaling Enhances the Antioxidant Capacity of Salt-Stressed Cucumber Roots. Plant Physiol. Biochem..

[140] Hongna C., Junmei S., Leyuan T., Xiaori H., Guolin L., Xianguo C. (2021). Exogenous Spermidine Priming Mitigates the Osmotic Damage in Germinating Seeds of *Leymus chinensis* under Salt-Alkali Stress. Front. Plant Sci..

[141] He M.W., Wang Y., Wu J.Q., Shu S., Sun J., Guo S.R. (2019). Isolation and Characterization of S-Adenosylmethionine Synthase Gene from Cucumber and Responsive to Abiotic Stress. Plant Physiol. Biochem..

[142] Li H., Liu S.S., Yi C.Y., Wang F., Zhou J., Xia X.J., Shi K., Zhou Y.H., Yu J.Q. (2014). Hydrogen Peroxide Mediates Abscisic Acid-Induced HSP70 Accumulation and Heat Tolerance in Grafted Cucumber Plants. Plant Cell Environ..

[143] Luo S., Liu Z., Wan Z., He X., Lv J., Yu J., Zhang G. (2023). Foliar Spraying of NaHS Alleviates Cucumber Salt Stress by Maintaining Na^+^/K^+^ Balance and Activating Salt Tolerance Signaling Pathways. Plants.

[144] Santos A.S., Almeida J.F., Silva M.S.D., Nóbrega J.S., Queiroga T.B.D., Pereira J.A.R., Linné J.A., Gomes F.A.L. (2019). The Influence of H_2_O_2_ Application Methods on Melon Plants Submitted to Saline Stress. J. Agric. Sci..

[145] Zhang H.J., Zhang N., Yang R.C., Wang L., Sun Q.Q., Li D.B., Cao Y.Y., Weeda S., Zhao B., Ren S. (2014). Melatonin Promotes Seed Germination under High Salinity by Regulating Antioxidant Systems, ABA and GA(4) Interaction in Cucumber (*Cucumis sativus* L.). J. Pineal Res..

[146] Sheikhalipour M., Mohammadi S.A., Esmaielpour B., Zareei E., Kulak M., Ali S., Nouraein M., Bahrami M.K., Gohari G., Fotopoulos V. (2022). Exogenous Melatonin Increases Salt Tolerance in Bitter Melon by Regulating Ionic Balance, Antioxidant System and Secondary Metabolism-Related Genes. BMC Plant Biol..

[147] Amerian M., Palangi A., Gohari G., Ntatsi G. (2024). Enhancing Salinity Tolerance in Cucumber through Selenium Biofortification and Grafting. BMC Plant Biol..

[148] Zhu L.J., Yan Q.J., Chen G.S., Hu J., Luo M., Yang Y. (2019). Exogenous H_2_O_2_ Promotes Seed Germination under High Salinity by Regulating Antioxidant Enzymes, ABA and GA Interaction in Cucumber (*Cucumis sativus*). Plant Physiol. J..

[149] Singh V.P., Srivastava P.K., Prasad S.M. (2013). Nitric Oxide Alleviates Arsenic-Induced Toxic Effects in Ridged Luffa Seedlings. Plant Physiol. Biochem..

[150] Feng L., Li Q., Zhou D., Jia M., Liu Z., Hou Z., Ren Q., Ji S., Sang S., Lu S. (2024). *B. subtilis* CNBG-PGPR-1 Induces Methionine to Regulate Ethylene Pathway and ROS Scavenging for Improving Salt Tolerance of Tomato. Plant J..

[151] Liang L., Tang W., Lian H., Sun B., Huang Z., Sun G., Li X., Tu L., Li H., Tang Y. (2022). Grafting Promoted Antioxidant Capacity and Carbon and Nitrogen Metabolism of Bitter Gourd Seedlings under Heat Stress. Front. Plant Sci..

[152] Wang Q., Shen T., Ni L., Chen C., Jiang J., Cui Z., Wang S., Xu F., Yan R., Jiang M. (2023). Phosphorylation of OsRbohB by the CaM-Dependent Protein Kinase OsDMI3 Promotes H_2_O_2_ Production to Potentiate ABA Responses in Rice. Mol. Plant.

[153] Qin R., Hu Y., Chen H., Du Q., Yang J., Li W.X. (2023). MicroRNA408 Negatively Regulates Salt Tolerance by Affecting Secondary Cell Wall Development in Maize. Plant Physiol..

[154] Deng Y., Liu S., Zhang Y., Tan J., Li X., Chu X., Xu B., Tian Y., Sun Y., Li B. (2022). A Telomere-to-Telomere Gap-Free Reference Genome of Watermelon and Its Mutation Library Provide Important Resources for Gene Discovery and Breeding. Mol. Plant.

[155] Wei M., Huang Y., Mo C., Wang H., Zeng Q., Yang W., Chen J., Zhang X., Kong Q. (2023). Telomere-to-Telomere Genome Assembly of Melon (*Cucumis melo* L. Var. Inodorus) Provides a High-Quality Reference for Meta-QTL Analysis of Important Traits. Hortic. Res..

